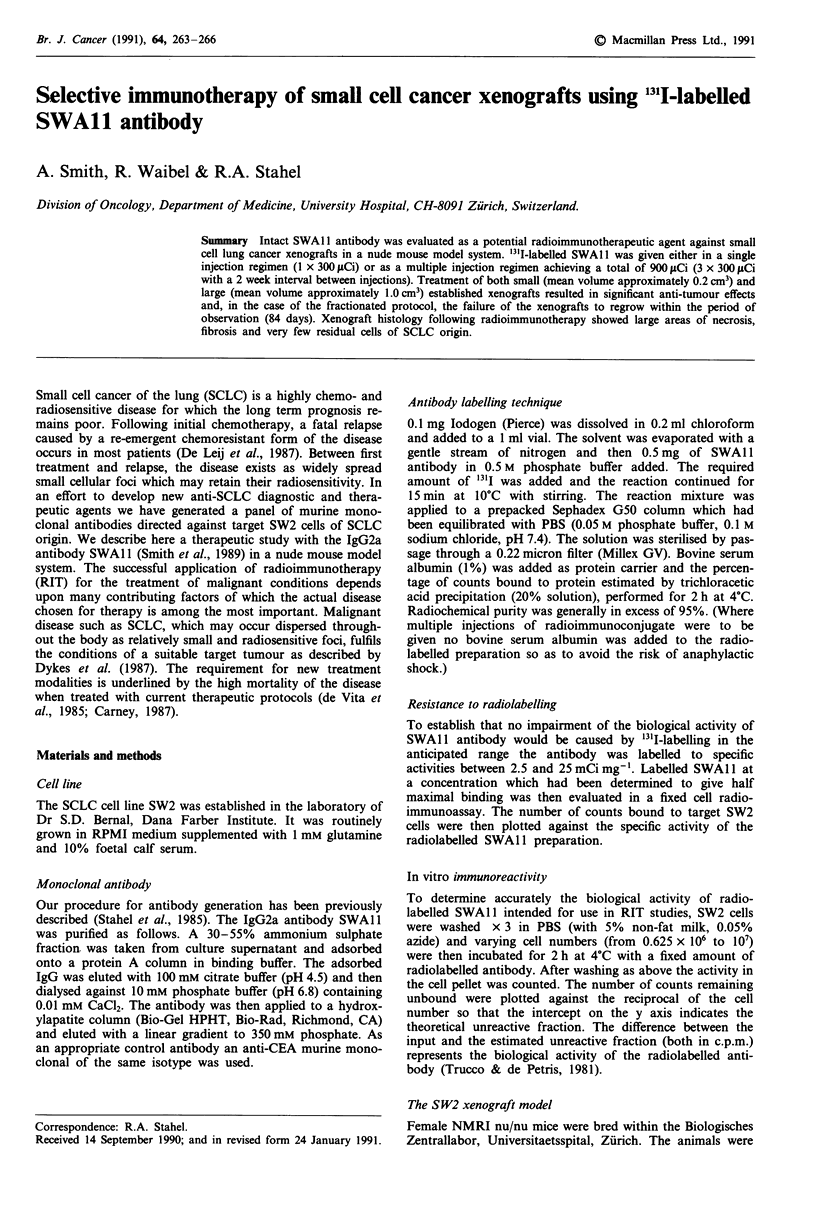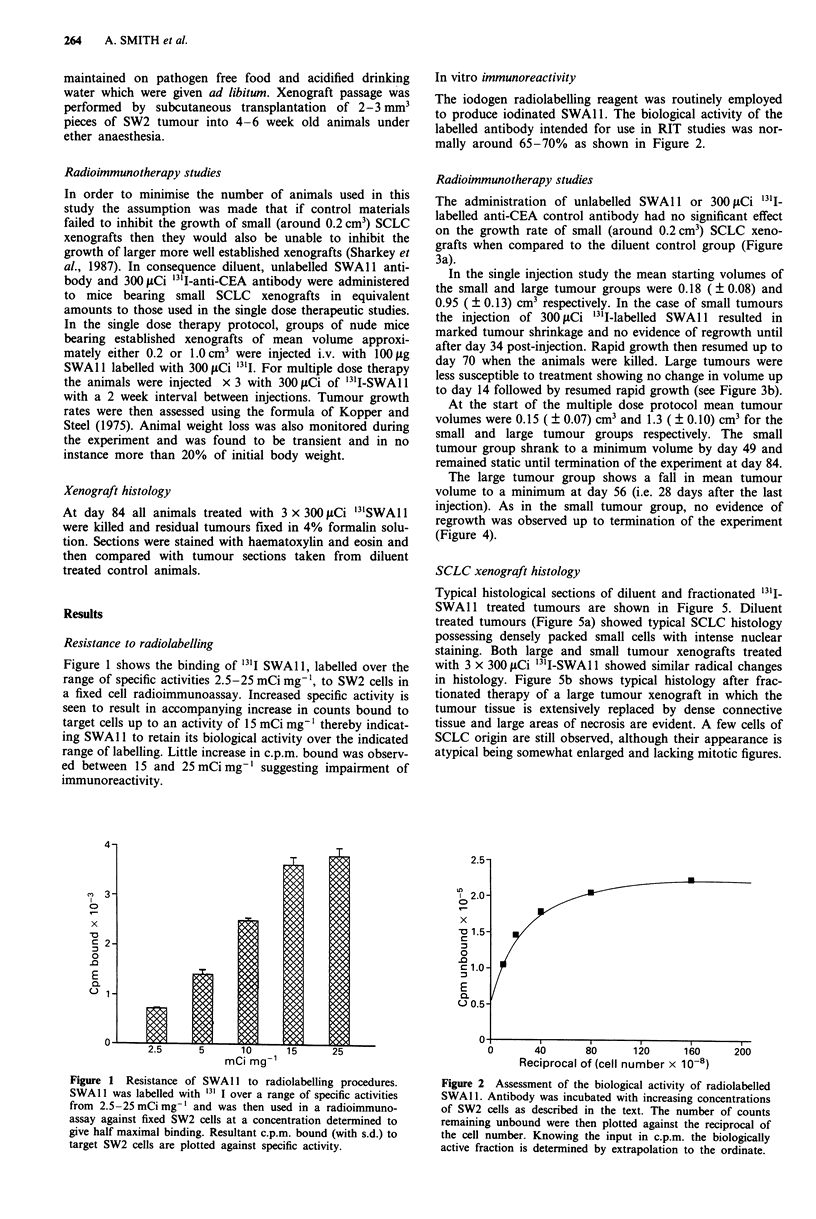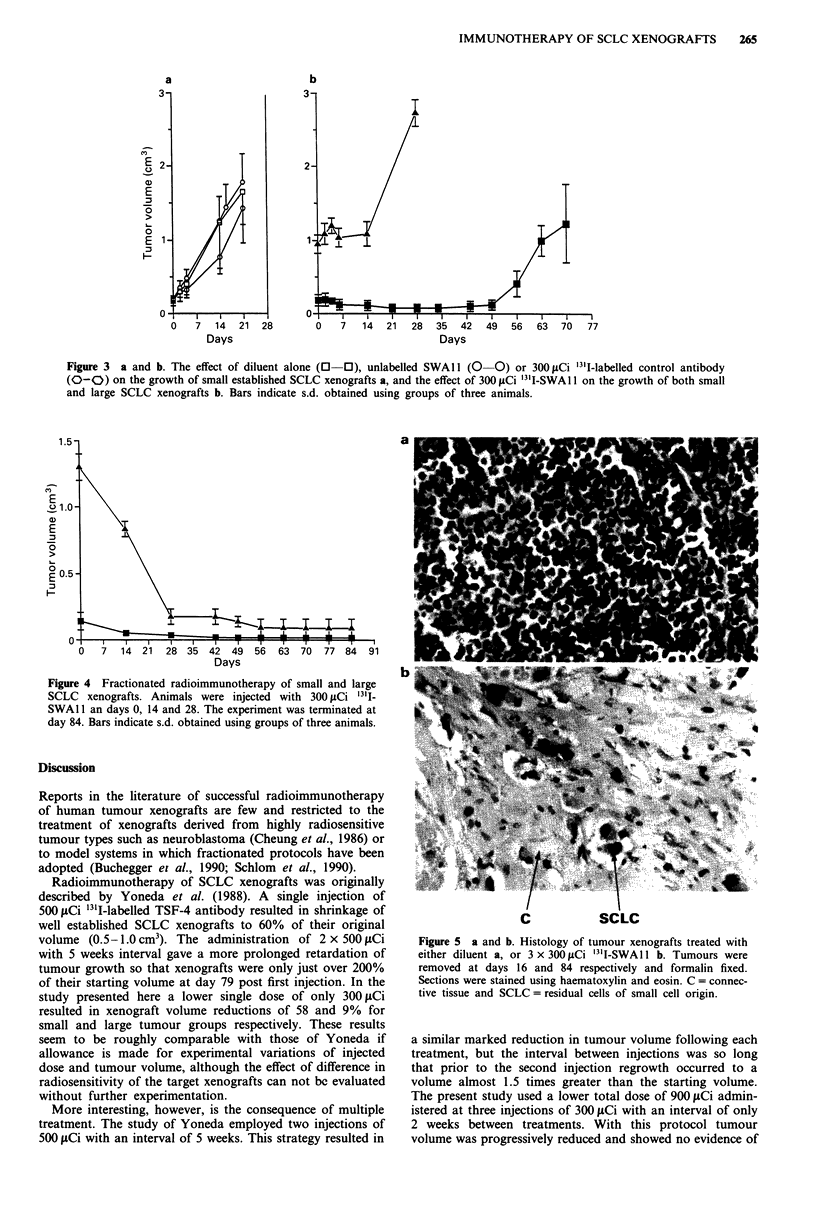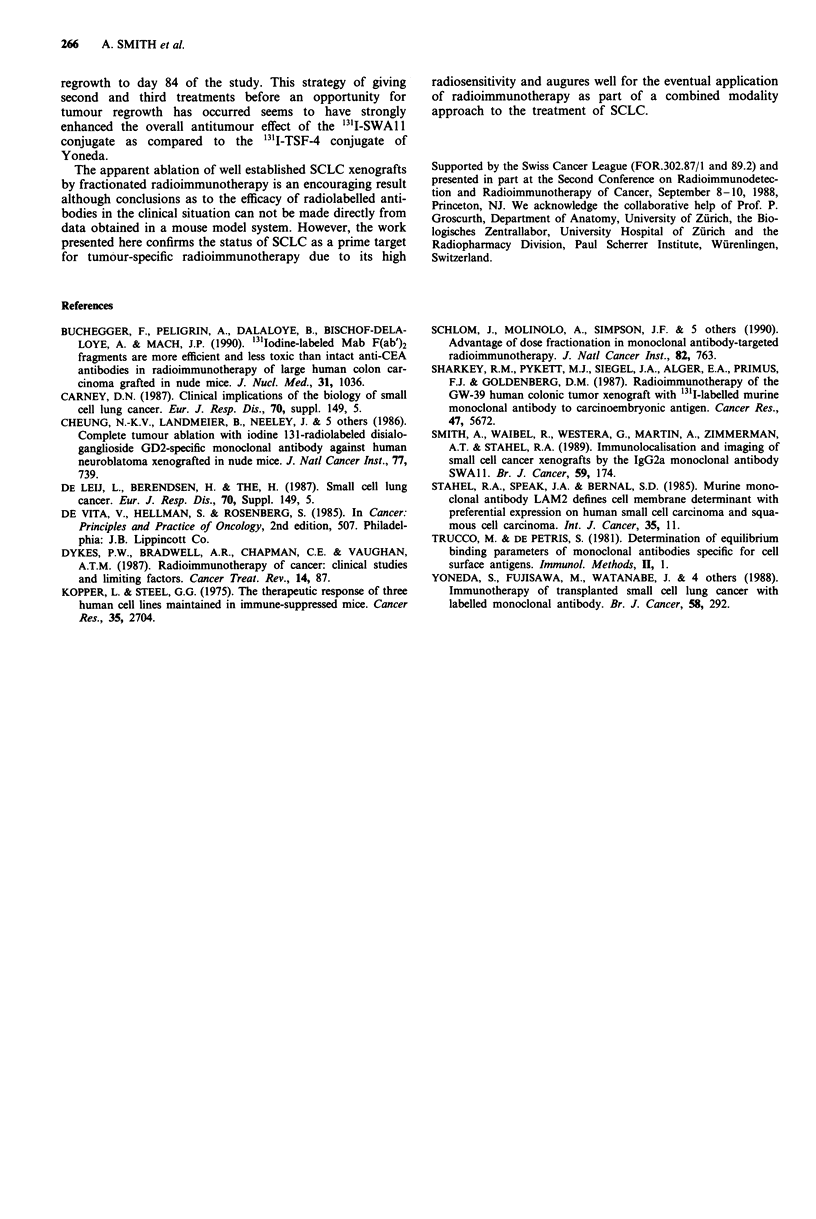# Selective immunotherapy of small cell cancer xenografts using 131I-labelled SWA11 antibody.

**DOI:** 10.1038/bjc.1991.289

**Published:** 1991-08

**Authors:** A. Smith, R. Waibel, R. A. Stahel

**Affiliations:** Department of Medicine, University Hospital, Zürich, Switzerland.

## Abstract

**Images:**


Br. J. Cancer (1991), 64, 263-266 © Macmillan Press Ltd., 1991

### Selective immunotherapy of small cell cancer xenografts using $^{131}$I-labelled SWA11 antibody

A. Smith, R. Waibel & R.A. Stahel

*Division of Oncology, Department of Medicine, University Hospital, CH-8091 Zürich, Switzerland.*

**Summary** Intact SWA11 antibody was evaluated as a potential radioimmunotherapeutic agent against small cell lung cancer xenografts in a nude mouse model system. $^{131}$I-labelled SWA11 was given either in a single injection regimen (1 × 300 μCi) or as a multiple injection regimen achieving a total of 900 μCi (3 × 300 μCi with a 2 week interval between injections). Treatment of both small (mean volume approximately 0.2 cm$^3$) and large (mean volume approximately 1.0 cm$^3$) established xenografts resulted in significant anti-tumour effects and, in the case of the fractionated protocol, the failure of the xenografts to regrow within the period of observation (84 days). Xenograft histology following radioimmunotherapy showed large areas of necrosis, fibrosis and very few residual cells of SCLC origin.

Small cell cancer of the lung (SCLC) is a highly chemo- and radiosensitive disease for which the long term prognosis remains poor. Following initial chemotherapy, a fatal relapse caused by a re-emergent chemoresistant form of the disease occurs in most patients (De Leij *et al.*, 1987). Between first treatment and relapse, the disease exists as widely spread small cellular foci which may retain their radiosensitivity. In an effort to develop new anti-SCLC diagnostic and therapeutic agents we have generated a panel of murine monoclonal antibodies directed against target SW2 cells of SCLC origin. We describe here a therapeutic study with the IgG2a antibody SWA11 (Smith *et al.*, 1989) in a nude mouse model system. The successful application of radioimmunotherapy (RIT) for the treatment of malignant conditions depends upon many contributing factors of which the actual disease chosen for therapy is among the most important. Malignant disease such as SCLC, which may occur dispersed throughout the body as relatively small and radiosensitive foci, fulfils the conditions of a suitable target tumour as described by Dykes *et al.* (1987). The requirement for new treatment modalities is underlined by the high mortality of the disease when treated with current therapeutic protocols (de Vita *et al.*, 1985; Carney, 1987).

### Materials and methods

### *Cell line*

The SCLC cell line SW2 was established in the laboratory of Dr S.D. Bernal, Dana Farber Institute. It was routinely grown in RPMI medium supplemented with 1 mM glutamine and 10% foetal calf serum.

### *Monoclonal antibody*

Our procedure for antibody generation has been previously described (Stahel *et al.*, 1985). The IgG2a antibody SWA11 was purified as follows. A 30–55% ammonium sulphate fraction was taken from culture supernatant and adsorbed onto a protein A column in binding buffer. The adsorbed IgG was eluted with 100 mM citrate buffer (pH 4.5) and then dialysed against 10 mM phosphate buffer (pH 6.8) containing 0.01 mM $CaCl_2$. The antibody was then applied to a hydroxylapatite column (Bio-Gel HPHT, Bio-Rad, Richmond, CA) and eluted with a linear gradient to 350 mM phosphate. As an appropriate control antibody an anti-CEA murine monoclonal of the same isotype was used.

### *Antibody labelling technique*

0.1 mg Iodogen (Pierce) was dissolved in 0.2 ml chloroform and added to a 1 ml vial. The solvent was evaporated with a gentle stream of nitrogen and then 0.5 mg of SWA11 antibody in 0.5 M phosphate buffer added. The required amount of $^{131}$I was added and the reaction continued for 15 min at 10°C with stirring. The reaction mixture was applied to a prepacked Sephadex G50 column which had been equilibrated with PBS (0.05 M phosphate buffer, 0.1 M sodium chloride, pH 7.4). The solution was sterilised by passage through a 0.22 micron filter (Millex GV). Bovine serum albumin (1%) was added as protein carrier and the percentage of counts bound to protein estimated by trichloracetic acid precipitation (20% solution), performed for 2 h at 4°C. Radiochemical purity was generally in excess of 95%. (Where multiple injections of radioimmunoconjugate were to be given no bovine serum albumin was added to the radiolabelled preparation so as to avoid the risk of anaphylactic shock.)

### *Resistance to radiolabelling*

To establish that no impairment of the biological activity of SWA11 antibody would be caused by $^{131}$I-labelling in the anticipated range the antibody was labelled to specific activities between 2.5 and 25 mCi mg$^{-1}$. Labelled SWA11 at a concentration which had been determined to give half maximal binding was then evaluated in a fixed cell radioimmunoassay. The number of counts bound to target SW2 cells were then plotted against the specific activity of the radiolabelled SWA11 preparation.

### In vitro *immunoreactivity*

To determine accurately the biological activity of radiolabelled SWA11 intended for use in RIT studies, SW2 cells were washed × 3 in PBS (with 5% non-fat milk, 0.05% azide) and varying cell numbers (from 0.625 × 10$^6$ to 10$^7$) were then incubated for 2 h at 4°C with a fixed amount of radiolabelled antibody. After washing as above the activity in the cell pellet was counted. The number of counts remaining unbound were plotted against the reciprocal of the cell number so that the intercept on the y axis indicates the theoretical unreactive fraction. The difference between the input and the estimated unreactive fraction (both in c.p.m.) represents the biological activity of the radiolabelled antibody (Trucco & de Petris, 1981).

### *The SW2 xenograft model*

Female NMRI nu/nu mice were bred within the Biologisches Zentrallabor, Universitaetsspital, Zürich. The animals were

Correspondence: R.A. Stahel.
Received 14 September 1990; and in revised form 24 January 1991.

maintained on pathogen free food and acidified drinking water which were given *ad libitum*. Xenograft passage was performed by subcutaneous transplantation of 2–3 $mm^3$ pieces of SW2 tumour into 4–6 week old animals under ether anaesthesia.

*Radioimmunotherapy studies*

In order to minimise the number of animals used in this study the assumption was made that if control materials failed to inhibit the growth of small (around 0.2 $cm^3$) SCLC xenografts then they would also be unable to inhibit the growth of larger more well established xenografts (Sharkey *et al.*, 1987). In consequence diluent, unlabelled SWA11 antibody and 300 μCi $^{131}$I-anti-CEA antibody were administered to mice bearing small SCLC xenografts in equivalent amounts to those used in the single dose therapeutic studies. In the single dose therapy protocol, groups of nude mice bearing established xenografts of mean volume approximately either 0.2 or 1.0 $cm^3$ were injected i.v. with 100 μg SWA11 labelled with 300 μCi $^{131}$I. For multiple dose therapy the animals were injected × 3 with 300 μCi of $^{131}$I-SWA11 with a 2 week interval between injections. Tumour growth rates were then assessed using the formula of Kopper and Steel (1975). Animal weight loss was also monitored during the experiment and was found to be transient and in no instance more than 20% of initial body weight.

*Xenograft histology*

At day 84 all animals treated with 3 × 300 μCi $^{131}$SWA11 were killed and residual tumours fixed in 4% formalin solution. Sections were stained with haematoxylin and eosin and then compared with tumour sections taken from diluent treated control animals.

### Results

*Resistance to radiolabelling*

Figure 1 shows the binding of $^{131}$I SWA11, labelled over the range of specific activities 2.5–25 mCi $mg^{-1}$, to SW2 cells in a fixed cell radioimmunoassay. Increased specific activity is seen to result in accompanying increase in counts bound to target cells up to an activity of 15 mCi $mg^{-1}$ thereby indicating SWA11 to retain its biological activity over the indicated range of labelling. Little increase in c.p.m. bound was observed between 15 and 25 mCi $mg^{-1}$ suggesting impairment of immunoreactivity.

*In vitro immunoreactivity*

The iodogen radiolabelling reagent was routinely employed to produce iodinated SWA11. The biological activity of the labelled antibody intended for use in RIT studies was normally around 65–70% as shown in Figure 2.

*Radioimmunotherapy studies*

The administration of unlabelled SWA11 or 300 μCi $^{131}$I-labelled anti-CEA control antibody had no significant effect on the growth rate of small (around 0.2 $cm^3$) SCLC xenografts when compared to the diluent control group (Figure 3a).

In the single injection study the mean starting volumes of the small and large tumour groups were 0.18 (± 0.08) and 0.95 (± 0.13) $cm^3$ respectively. In the case of small tumours the injection of 300 μCi $^{131}$I-labelled SWA11 resulted in marked tumour shrinkage and no evidence of regrowth until after day 34 post-injection. Rapid growth then resumed up to day 70 when the animals were killed. Large tumours were less susceptible to treatment showing no change in volume up to day 14 followed by resumed rapid growth (see Figure 3b).

At the start of the multiple dose protocol mean tumour volumes were 0.15 (± 0.07) $cm^3$ and 1.3 (± 0.10) $cm^3$ for the small and large tumour groups respectively. The small tumour group shrank to a minimum volume by day 49 and remained static until termination of the experiment at day 84.

The large tumour group shows a fall in mean tumour volume to a minimum at day 56 (i.e. 28 days after the last injection). As in the small tumour group, no evidence of regrowth was observed up to termination of the experiment (Figure 4).

*SCLC xenograft histology*

Typical histological sections of diluent and fractionated $^{131}$I-SWA11 treated tumours are shown in Figure 5. Diluent treated tumours (Figure 5a) showed typical SCLC histology possessing densely packed small cells with intense nuclear staining. Both large and small tumour xenografts treated with 3 × 300 μCi $^{131}$I-SWA11 showed similar radical changes in histology. Figure 5b shows typical histology after fractionated therapy of a large tumour xenograft in which the tumour tissue is extensively replaced by dense connective tissue and large areas of necrosis are evident. A few cells of SCLC origin are still observed, although their appearance is atypical being somewhat enlarged and lacking mitotic figures.

**Figure 1** Resistance of SWA11 to radiolabelling procedures. SWA11 was labelled with $^{131}$ I over a range of specific activities from 2.5–25 mCi $mg^{-1}$ and was then used in a radioimmunoassay against fixed SW2 cells at a concentration determined to give half maximal binding. Resultant c.p.m. bound (with s.d.) to target SW2 cells are plotted against specific activity.

**Figure 2** Assessment of the biological activity of radiolabelled SWA11. Antibody was incubated with increasing concentrations of SW2 cells as described in the text. The number of counts remaining unbound were then plotted against the reciprocal of the cell number. Knowing the input in c.p.m. the biologically active fraction is determined by extrapolation to the ordinate.

**Figure 3** a and b. The effect of diluent alone (□—□), unlabelled SWA11 (O—O) or 300 μCi $^{131}$I-labelled control antibody (O–O) on the growth of small established SCLC xenografts a, and the effect of 300 μCi $^{131}$I-SWA11 on the growth of both small and large SCLC xenografts b. Bars indicate s.d. obtained using groups of three animals.

**Figure 4** Fractionated radioimmunotherapy of small and large SCLC xenografts. Animals were injected with 300 μCi $^{131}$I-SWA11 an days 0, 14 and 28. The experiment was terminated at day 84. Bars indicate s.d. obtained using groups of three animals.

### Discussion

Reports in the literature of successful radioimmunotherapy of human tumour xenografts are few and restricted to the treatment of xenografts derived from highly radiosensitive tumour types such as neuroblastoma (Cheung *et al.*, 1986) or to model systems in which fractionated protocols have been adopted (Buchegger *et al.*, 1990; Schlom *et al.*, 1990).

Radioimmunotherapy of SCLC xenografts was originally described by Yoneda *et al.* (1988). A single injection of 500 μCi $^{131}$I-labelled TSF-4 antibody resulted in shrinkage of well established SCLC xenografts to 60% of their original volume (0.5–1.0 cm$^3$). The administration of 2 × 500 μCi with 5 weeks interval gave a more prolonged retardation of tumour growth so that xenografts were only just over 200% of their starting volume at day 79 post first injection. In the study presented here a lower single dose of only 300 μCi resulted in xenograft volume reductions of 58 and 9% for small and large tumour groups respectively. These results seem to be roughly comparable with those of Yoneda if allowance is made for experimental variations of injected dose and tumour volume, although the effect of difference in radiosensitivity of the target xenografts can not be evaluated without further experimentation.

More interesting, however, is the consequence of multiple treatment. The study of Yoneda employed two injections of 500 μCi with an interval of 5 weeks. This strategy resulted in a similar marked reduction in tumour volume following each treatment, but the interval between injections was so long that prior to the second injection regrowth occurred to a volume almost 1.5 times greater than the starting volume. The present study used a lower total dose of 900 μCi administered at three injections of 300 μCi with an interval of only 2 weeks between treatments. With this protocol tumour volume was progressively reduced and showed no evidence of

**Figure 5** a and b. Histology of tumour xenografts treated with either diluent a, or 3 × 300 μCi $^{131}$I-SWA11 b. Tumours were removed at days 16 and 84 respectively and formalin fixed. Sections were stained using haematoxylin and eosin. C = connective tissue and SCLC = residual cells of small cell origin.

regrowth to day 84 of the study. This strategy of giving second and third treatments before an opportunity for tumour regrowth has occurred seems to have strongly enhanced the overall antitumour effect of the $^{131}$I-SWA11 conjugate as compared to the $^{131}$I-TSF-4 conjugate of Yoneda.

The apparent ablation of well established SCLC xenografts by fractionated radioimmunotherapy is an encouraging result although conclusions as to the efficacy of radiolabelled antibodies in the clinical situation can not be made directly from data obtained in a mouse model system. However, the work presented here confirms the status of SCLC as a prime target for tumour-specific radioimmunotherapy due to its high radiosensitivity and augures well for the eventual application of radioimmunotherapy as part of a combined modality approach to the treatment of SCLC.

Supported by the Swiss Cancer League (FOR.302.87/1 and 89.2) and presented in part at the Second Conference on Radioimmunodetection and Radioimmunotherapy of Cancer, September 8–10, 1988, Princeton, NJ. We acknowledge the collaborative help of Prof. P. Groscurth, Department of Anatomy, University of Zürich, the Biologisches Zentrallabor, University Hospital of Zürich and the Radiopharmacy Division, Paul Scherrer Institute, Würenlingen, Switzerland.

### References

BUCHEGGER, F., PELIGRIN, A., DALALOYE, B., BISCHOF-DELALOYE, A. & MACH, J.P. (1990). $^{131}$Iodine-labeled Mab F(ab')$_2$ fragments are more efficient and less toxic than intact anti-CEA antibodies in radioimmunotherapy of large human colon carcinoma grafted in nude mice. *J. Nucl. Med.*, **31**, 1036.

CARNEY, D.N. (1987). Clinical implications of the biology of small cell lung cancer. *Eur. J. Resp. Dis.*, **70**, suppl. 149, 5.

CHEUNG, N.-K.V., LANDMEIER, B., NEELEY, J. & 5 others (1986). Complete tumour ablation with iodine 131-radiolabeled disialoganglioside GD2-specific monoclonal antibody against human neuroblatoma xenografted in nude mice. *J. Natl Cancer Inst.*, **77**, 739.

DE LEIJ, L., BERENDSEN, H. & THE, H. (1987). Small cell lung cancer. *Eur. J. Resp. Dis.*, **70**, Suppl. 149, 5.

DE VITA, V., HELLMAN, S. & ROSENBERG, S. (1985). In *Cancer: Principles and Practice of Oncology*, 2nd edition, 507. Philadelphia: J.B. Lippincott Co.

DYKES, P.W., BRADWELL, A.R., CHAPMAN, C.E. & VAUGHAN, A.T.M. (1987). Radioimmunotherapy of cancer: clinical studies and limiting factors. *Cancer Treat. Rev.*, **14**, 87.

KOPPER, L. & STEEL, G.G. (1975). The therapeutic response of three human cell lines maintained in immune-suppressed mice. *Cancer Res.*, **35**, 2704.

SCHLOM, J., MOLINOLO, A., SIMPSON, J.F. & 5 others (1990). Advantage of dose fractionation in monoclonal antibody-targeted radioimmunotherapy. *J. Natl Cancer Inst.*, **82**, 763.

SHARKEY, R.M., PYKETT, M.J., SIEGEL, J.A., ALGER, E.A., PRIMUS, F.J. & GOLDENBERG, D.M. (1987). Radioimmunotherapy of the GW-39 human colonic tumor xenograft with $^{131}$I-labelled murine monoclonal antibody to carcinoembryonic antigen. *Cancer Res.*, **47**, 5672.

SMITH, A., WAIBEL, R., WESTERA, G., MARTIN, A., ZIMMERMAN, A.T. & STAHEL, R.A. (1989). Immunolocalisation and imaging of small cell cancer xenografts by the IgG2a monoclonal antibody SWA11. *Br. J. Cancer*, **59**, 174.

STAHEL, R.A., SPEAK, J.A. & BERNAL, S.D. (1985). Murine monoclonal antibody LAM2 defines cell membrane determinant with preferential expression on human small cell carcinoma and squamous cell carcinoma. *Int. J. Cancer*, **35**, 11.

TRUCCO, M. & DE PETRIS, S. (1981). Determination of equilibrium binding parameters of monoclonal antibodies specific for cell surface antigens. *Immunol. Methods*, **II**, 1.

YONEDA, S., FUJISAWA, M., WATANABE, J. & 4 others (1988). Immunotherapy of transplanted small cell lung cancer with labelled monoclonal antibody. *Br. J. Cancer*, **58**, 292.